# β-FeOOH Interlayer With Abundant Oxygen Vacancy Toward Boosting Catalytic Effect for Lithium Sulfur Batteries

**DOI:** 10.3389/fchem.2020.00309

**Published:** 2020-04-23

**Authors:** Yingying Li, Xifei Li, Youchen Hao, Alibek Kakimov, Dejun Li, Qian Sun, Liang Kou, Zhanyuan Tian, Le Shao, Cheng Zhang, Jiujun Zhang, Xueliang Sun

**Affiliations:** ^1^Tianjin International Joint Research Centre of Surface Technology for Energy Storage Materials, Energy & Materials Engineering Centre, College of Physics and Materials Science, Tianjin Normal University, Tianjin, China; ^2^Xi'an Key Laboratory of New Energy Materials and Devices, Institute of Advanced Electrochemical Energy & School of Materials Science and Engineering, Xi'an University of Technology, Xi'an, China; ^3^State Center for International Cooperation on Designer Low-Carbon & Environmental Materials (CDLCEM), Zhengzhou University, Zhengzhou, China; ^4^Department of Mechanical and Materials Engineering, University of Western Ontario, London, ON, Canada; ^5^Shaanxi Coal Chemical Industry Technology Research Institute Co., Ltd., Xi'an, China; ^6^Department of Chemistry, College of Sciences/Institute for Sustainable Energy, Shanghai University, Shanghai, China

**Keywords:** CNTs@FeOOH, oxygen vacancy, catalytic effect, polysulfides, Li-S batteries

## Abstract

Due to the shuttle effect and low conductivity of sulfur (S), it has been challenging to realize the application of lithium-sulfur (Li-S) batteries with high performance and long cyclability. In this study, a high catalytic active CNTs@FeOOH composite is introduced as a functional interlayer for Li-S batteries. Interestingly, the existence of oxygen vacancy in FeOOH functions electrocatalyst and promotes the catalytic conversion of intercepted lithium polysulfides (LiPS). As a result, the optimized CNTs@FeOOH interlayer contributed to a high reversible capacity of 556 mAh g^−1^ at 3,200 mA g^−1^ over 350 cycles. This study demonstrates that enhanced catalytic effect can accelerate conversion efficiency of polysulfides, which is beneficial of boosting high performance Li-S batteries.

## Introduction

Currently, Li-S batteries have received extensive attention due to their high theoretical capacity (1,675 mAh g^−1^), high energy density (2,600 Wh kg^−1^) (Ji and Nazar, 2010; Manthiram et al., 2013), low cost and environmental friendliness (Nazar et al., 2014; Yang et al., 2018). Considering the low conductivity of S (σ = 5.0 × 10^−30^ S cm^−1^) and the shuttle effect of LiPS, research on Li-S batteries is strongly delayed and thus hardly meets actual needs (Zhang, 2013; Rosenman et al., 2015).

To solve these problems, many pioneering works are using a porous carbon-based host, a functional interlayer, absorptable polar composites [such as CoS_2_/C (Yuan et al., 2016), MnO_2_/GO/CNT (Kong et al., 2017), S@TiO_2_ (Wei Seh et al., 2013) etc.] and a catalytic effect on LiPS conversion. Among them, carbon-based hybrid materials with absorbility to LiPS have always shown attractive characteristics (Tang and Hou, 2018). For instance, Pang and Nazar obtained C_3_N_4_ by pyrolysis of melamine, which has rich pyridine nitrogen adsorption sites. This can lead to the fact that the sulfur electrode with ultra-low long-term capacity fades out by 0.04% for a cycle over 1,500 cycles at a practical rate of 0.5C (Pang and Nazar, 2016). However, it should be noted that absorbed LiPS is easy to release, because these materials are soluble in the electrolyte. Thus, a more efficient strategy is urgently needed to meet this problem. Catalyst has been employed to accelerate the transformation of LiPS from liquid to solid, which corresponds to the transmission from long-chain Li_2_S_6_ to short-chain Li_2_S. We strongly believe that this is an alternative option to modify Li-S batteries by introducing catalytic materials to catalyze the conversion of LiPS into insoluble products. Yang group reported the Fe_3_C/Fe-N_x_@NPCN modified separator, due to the catalytic effect of Fe_3_C to LiPS, the modified batteries yielded a high capacity of 1,517 mAh g^−1^ at 0.1C and displayed a capacity decay rate of 0.034% per cycle after 500 cycles at 1C (Yang et al., 2019). Bian et al. took multi-functional porous carbon nanofibers (g-C_3_N_4_@PCNF) as the sulfur host, in which g-C_3_N_4_ contributed to rapid oxidation-remediation conversion of S species and slowed down LiPS yield. Consequently, the g-C_3_N_4_@PCNF/S cathode achieves good flexibility and excellent cycling retention, e.g., long cycling with decay power of only 0.056% per cycle for 500 cycles at 1.0 A g^−1^ (Bian et al., 2019). In addition, Lee and coworkers have demonstrated that catalytic activity in anoxic sites is higher than in saturated sites as the oxygen vacancy can contribute to the transformation of electrons and the formation of S^3−^ radicals (Lin et al., 2018). Therefore, we are inspired to develop more powerful carbon-based composites with better catalytic effect for LiPS.

As previously reported, FeOOH with abundant oxygen vacancies (Zhang et al., 2018) can be a promising candidate as the catalyst for LiPS. We propose using CNTs@FeOOH composite materials to enhance the stability of Li-S batteries. On the one hand, FeOOH is efficient to catalyze the transformation of polysulfides; on the other hand, CNTs provide a fast electron transport channel, which ensures the sustainability of the reaction under high current and reduces the occurrence of polarization. As a result, the obtained electrodes maintained a high reversible capacity of 556 mAh g^−1^ at 2C for 350 cycles. This work plays a significant contribution to the development of Li-S batteries with high performance and long lifespan.

## Experimental Section

### Synthesis of CNTs@FeOOH Composite and Separator

As reported in our previous work (Hao et al., 2019), CNTs@FeOOH composites with various mass ratio can be obtained by controlling the content of iron source. For comparison, the pure FeOOH phase was synthesized under the same conditions without adding CNTs.

CNTs@FeOOH composites were homogenized into paste by N, N-2-methyl pyrrolidone, the slurry was evenly coated on the polypropylene separator (PP separator). The CNTs@FeOOH-coated separator was dried overnight in vacuum at 40°C and then cut into circular disks (16 mm). CNTs and pure FeOOH phase were wrapped on the separator surface by the same method as the control experiment.

### Preparation of the Pure Sulfur Cathode

The commercial S powder, acetylene black and polyvinylidene fluoride binder (PVDF) with a mass ratio of 55:30:15 were mixed into N-methyl-2-pyrrolidone (NMP) solvent and stirred on an electromagnetic stirrer for 24 h to obtain the slurry. The slurry was then cast on Al foil and dried overnight in a vacuum oven at 60°C. The load mass of S is about 0.8 mg cm^−2^. And the lithium foil was used as the anode electrode.

### Materials Characterization

X-ray diffraction (XRD) spectra were tested by Bruker AXS D8 Advance diffractometer with Cu/Kα radiation. Thermogravimetric analysis (TGA, Pyris Diamond6000 TG/DTA, PerkinElmer Co., America) was performed to confirm the FeOOH content in composites. The morphologies of as-obtained samples were measured by a field-emission scanning electron microscope (SEM Hitach SU8010) and JEOLJEM-3000F transmission electron microscope (TEM). The composition of the elements on the composites surface was verified by X-ray photoelectron spectroscopy (XPS PHI5000 Versa Probe).

### Electrochemical Characterizations

Electrochemical performance was studied using CR2032 coin-typed cells assembled in an argon filled glovebox. 1.0 M lithium bis-trifluoromethanesulfonylimide (LiTFSI) in 1,3-dioxolane (DOL) and 1,2-dimethoxyethane (DME) at a volume ratio of 1: 1 with 1 wt% LiNO_3_ additive was utilized as electrolyte. Galvanostatic charge/discharge characteristics were tested on the LAND CT2001A battery tester. Princeton Applied Research Versa STAT4 was used to perform cyclic voltammetry at a scanning rate of 0.1 mV s^−1^. Electrochemical impedance spectroscopy (EIS) was performed on Princeton Applied Research Versa STAT4. The frequency range was 0.01 Hz−100 kHz amplitude of AC was 5.0 mV. All electrochemical tests were performed in the range of 1.7–2.8 V.

## Results and Discussion

**Figure 1:**
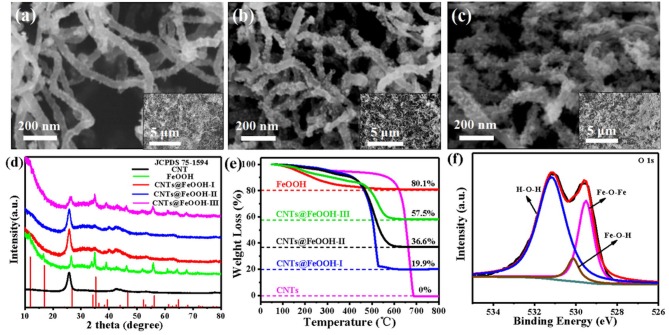
SEM images of **(a)** CNTs@FeOOH-I, **(b)** CNTs@FeOOH-II, **(c)** CNTs@FeOOH-III; **(d)** XRD, and **(e)** TGA of carboxylic CNTs, bare FeOOH and CNTs@FeOOH compounds with different proportions; **(f)** XPS spectra results of CNTs@FeOOH-II compounds: O 1s core-level spectrum.

The morphologies of CNTs, bare FeOOH and CNTs@FeOOH composites were visualized via scanning electron microscope (SEM). As depicted in Figures 1a–c, an increasingly obvious stick-like FeOOH being grown on the surface of CNTs (denoted as CNTs@FeOOH-I, II, and III, respectively). Compared to pure CNTs and FeOOH in Figure S1, the formation of rod-shaped FeOOH may induced by the reaction conditions. In addition, the X-ray diffraction (XRD) patterns of all samples are compared in Figure 1d. The peaks of pure FeOOH are located at 2θ = 11.925°, 16.901°, 26.874°, 34.185°, 35.311°, 39.386°, 46.656°, 52.349°, 56.158°, 61.278°, 64.718°, and 68.117° are attributed to the (110), (200), (130), (400), (211), (301), (411), (600), (251), (002), (541), and (132) reflection planes of β-FeOOH (JCPDS 75-1594). The FeOOH peaks become more evident with increasing FeOOH content and no impurity phase is detected. Transmission electron microscope (TEM) images of CNTs@FeOOH-II, demonstrated that FeOOH particles are uniformly adhered on the surface of CNTs, which consistent well with the results of SEM (see Figures S2a,b). The selected area electron diffraction pattern of the CNTs@FeOOH-II nanomaterials shows diffraction rings characteristic of FeOOH and CNTs (see Figure S2c), while the image of high resolution TEM in Figure S2d indicates lattice fringes corresponding to (110), (200), (211), and (330) planes. The results are consistent with those of XRD spectrums. Besides, thermogravimetric analysis (TGA) was used to calculate the proportion of components in various composites (Zhang et al., 2017). As shown in Figure 1e, the FeOOH content is measured as 24.8, 45.7, and 71.5 wt% for the composites CNTs@FeOOH-I, II, and III, respectively.

X-ray photoelectron spectroscopy (XPS) was employed to investigate the composition of elements on the surface of CNTs@FeOOH nanocomposites and the chemical states of various bond elements. The result shown the presence of Fe, O, and C atoms at the CNTs@FeOOH sheet surface (Figure S3a). The binding energy peak observed in the high-resolution C 1s profile at 284.9 eV (Figure S3b) can be attributed to graphite carbon in carbon nanotubes. The peak at 285.4 eV belongs to epoxy and hydroxyl (Beamson et al., 1994). The other two peaks are caused by carbonyl (C=O, 288.7 eV) and the oxygenated carbons of carboxyl (O-C=O, 291.2 eV) (Gardella et al., 1986; Kokai, 1990). These peaks reveal the existence of oxygen-containing functional groups on the surface of CNTs (Zhang et al., 2017). Meanwhile, the finescanned Fe 2p XPS spectra of that sample was also shown in Figure S3c, and the Fe 2p3/2 and Fe 2p1/2 peaks located at 711.0 and 724.9 eV could be indexed to Fe^3+^ and Fe^2+^, respectively, and satellite peaks at 719.4 and 733.9 eV correspond well with FeOOH (Tan et al., 1990). In addition, the O 1s peaks (Figure 1f) can be assigned to Fe-O-Fe (529.5 eV), Fe-O-H (530.1 eV), and H-O-H bonds (531.2 eV). It is worthwhile to point out that the peak at 531.2 eV is attributed to defect sites with low oxygen coordination (Zhang et al., 2019). According to previous reports, surface oxygen vacancies are involved in the LiPS transformation reaction, which significantly improves the kinetics of the reaction, thus contributing to the fast LiPS transformation at high rate.

**Figure 2:**
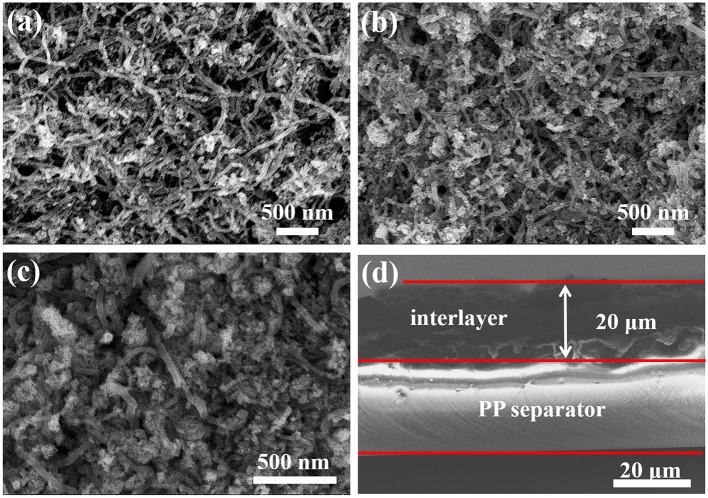
Surface morphology of **(a)** CNTs@FeOOH-I, **(b)** CNTs@FeOOH-II, **(c)** CNTs@FeOOH-III, and **(d)** cross section of CNTs@FeOOH-II interlayer.

According to the previous paper, the shuttle effect of polysulfides can be diminished with an additional intermediate layer (Fan et al., 2019). In this work, various composites of CNTs@FeOOH were coated on the surface of the PP separator as a functional interlayer to study their effect on Li-S batteries. Figure S4a shows a schematic representation of conventional PP-separator Li-S structures and advanced Li-S batteries with functional CNTs@FeOOH layers. The surface morphology of the modified interlayer is shown in Figures 2a–c, the preserved porous structure will facilitate electrolyte penetration and lithium ions (Li^+^) transfer. Figure 2d and Figures S4b,c show the cross-sectional appearance, and the thickness of the interlayer is about 20 um.

Coin-typed cells with different separators were assembled to evaluate electrochemical performances. Figure S4d exhibits the cycling performance of all Li-S cells with PP separator, CNTs, pure FeOOH and three proportional composites interlayer at 0.2C (1C = 1,675 mA g^−1^) between 1.7 and 2.8 V. After 100 cycles, CNTs@FeOOH-II revealed the best cyclability of 662.1 mAh g^−1^, which indicates a good synergistic effect between CNTs and FeOOH.

**Figure 3:**
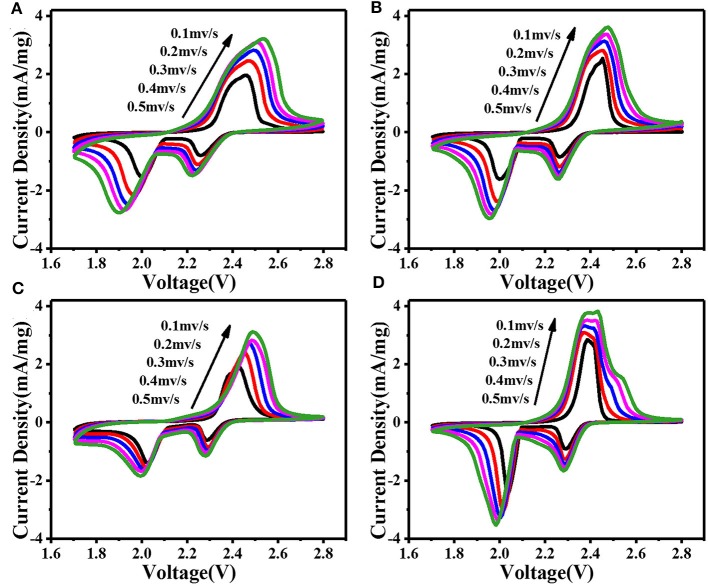
CV curves at various scan rates: the battery with **(A)** PP, **(B)** CNTs, **(C)** FeOOH, and **(D)** CNTs@FeOOH-II separator, respectively.

In order to explore the lithium diffusion properties and investigate the role of composite materials in Li-S batteries, we performed cyclic voltammetry (CV) measurements under various scanning rates ranging from 0.1 to 0.5 mV s^−1^ between 1.7 and 2.8 V (vs. Li/Li^+^). As shown in Figures 3A–D, all curves show typical reduction/oxidation reaction of S cathode, with two distinct cathode peaks and one anode peak. The cathode peak at about 2.3 V corresponds to the transformation of sulfur bonding with Li^+^ into soluble long-chain polysulfide [Li_2_S_x_ (x = 4–8)]. Furthermore, the cathode peak around 2.0 V corresponds to the transformation of long-chain polysulfide into insoluble Li_2_S or Li_2_*S*_2_ (Chung et al., 2016). In subsequent anode scanning, the oxidation peak at ~2.4 V corresponds to its reverse process. According to the relationship between CV scanning rate (ν^0.5^) and peak current (Ip), the lithium diffusion performance can be estimated using the classical Randles Sevcik equation:

(1) $$Ip=(2.69\times 10^{5})n^{1.5}SD_{\text{Li+}}^{\;0.5}\;C_{\text{Li}}\upsilon^{0.5}$$

where, Ip-peak current (A), *n*-number of electrons per type of reaction (*n* = 1), S-electrode area (S = 1.13 cm^2^), D_Li+_-diffusion coefficient of lithium ion (cm^2^ s^−1^), C_Li+_-initial concentration of lithium ion in the cathode (C_Li+_ = 1 mol cm^−3^), ν-potential scanning rate (V s^−1^) (Tao et al., 2016). The *n*, S, and C_Li+_ are constant in our battery system. The slope of the curve in Figure S5a is positively correlated with the corresponding Li^+^ diffusion. The calculated results show that the modified CNTs@FeOOH-II had the greatest diffusion capacity of Li 4.40 × 10^−12^, better than intact (2.08 × 10^−12^). Typically, the PP separator has difficulty catching soluble LiPS, which tends to dissolve in electrolytes in large quantities. As a result increasing viscosity of electrolyte leads to slower diffusion of Li^+^. On the contrary, since CNTs@FeOOH-II material can accelerate the conversion of LiPS to Li_2_*S*_2_ or Li_2_S, it is easier to increase the diffusion rate of Li^+^ in modified cells.

The discharge process in Li-S batteries can be expressed as follows:

(2) $${\text{S}}_{8}+{2\text{e}}^{-}↔{\text{S}}_{8}^{2-}$$

(3) $$3{\text{S}}_{8}+2{\text{e}}^{-}↔4{\text{S}}_{6}^{2-}$$

(4) $$2{\text{S}}_{6}^{2-}+2{\text{e}}^{-}↔3{\text{S}}_{4}^{2-}$$

(5) $${\text{S}}_{4}^{2-}+4{\text{Li}}^{+}+2{\text{e}}^{-}↔2{\text{Li}}_{2}{\text{S}}_{2}$$

(6) $${\text{Li}}_{2}{\text{S}}_{2}+2{\text{Li}}^{+}+{2\text{e}}^{-}↔2{\text{Li}}_{2}\text{S}$$

The theoretical discharge capacity of Li-S battery at different stages is calculated by referring the number of electrons transferred. The details are given in Table 1 and specific formulas are given below (Diao et al., 2013):

(7) $$\begin{array}{l} \text{q}=\text{nF}/\text{M} \end{array}$$

**Table 1:** The relationship between DOD and the discharge specific capacity.

**Discharge products**	**Transfer electron number/*n* (mol∙mol^**−1**^ S)**	**Depth of discharge DOD**	**Discharge specific capacity/q (mAh g^**−1**^)**
${\text{S}}_{8}\to {\text{S}}_{8}^{2-}$	0.25	12.5%	210
${\text{S}}_{8}\to {\text{S}}_{6}^{2-}$	0.33	16.7%	280
${\text{S}}_{8}\to {\text{S}}_{4}^{2-}$	0.5	25.0%	420
S_8_ → Li_2_S_2_	1	50.0%	840
S_8_ → Li_2_S	2	100.0%	1,680

**Figure 4:**
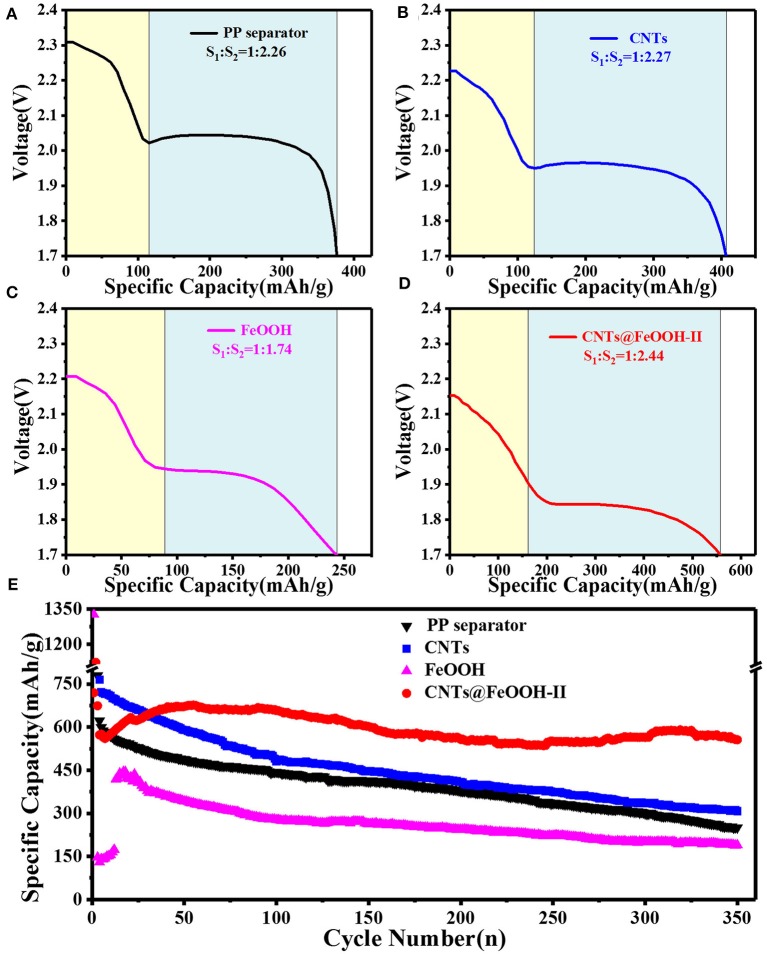
The 200th cycle Discharge profiles of the battery with **(A)** PP separator, **(B)** CNTs, **(C)** FeOOH, and **(D)** CNTs@FeOOH-II separator, respectively, at 3,200 mAh g^−1^; **(E)** cycle performance at 3,200 mAh g^−1^.

Among them, q is the specific discharge capacity, mAh g^−1^; n is the number of transfer electrons per mole mass, mol^−1^; F is the amount of electricity owned by 1 M electrons, 26.8 Ah; M is the molar mass of elemental sulfur, 32 g mol^−1^ (Diao et al., 2013). Here, the discharge capacity of ${\text{S}}_{8}\to S_{4}^{2-}$ is recorded as S_1_, and that of S_8_→*Li*_2_S as S_2_. Accordingly, S_1_:*S*_2_ is approach to 1:3. Figures 4A–D shows the discharge curve of the PP separator and various barrier interlayers circulating for 200 cycles at 2C. The calculated results show that the capacity ratio of CNTs@FeOOH-II (1:2.44) is closest to the theoretical value, higher than 1:2.26, 1:2.27, and 1:1.74 of PP separator, CNTs and FeOOH, respectively, which indicates the enhanced transformation ability of CNTs@FeOOH-II to polysulfide ions at high current density.

Figure 4E compares the cyclic characteristics of the batteries with PP separator, CNTs, pure phase FeOOH and CNTs@FeOOH-II separator in a voltage range of 1.7–2.8 V at 2C. The initial discharge capacities are 870.6, 1,093, 1326.4, and 1121.9 mAh g^−1^, respectively, show that CNTs@FeOOH-II interlayer can strengthen the utilization of S upon cycling. After 350 cycles, CNTs@FeOOH-II maintained a high reversible capacity of 556 mAh g^−1^. In detail, although CNTs have good electrical conductivity, the weak van der Waals interactions between polar LiPS and non-polar carbon materials results in slow release of S-active substances from carbon materials and obvious capacity decay during long cycle (Song et al., 2016). Simultaneously, it is remarkable that the highest reversible capacity of 1326.4 mAh g^−1^ can be obtained for pure FeOOH group. This can be explained by the presence of oxygen vacancies in FeOOH, which makes it electrocatalytic and prompts the rapid conversion of long-chain LiPS to solid Li_2_*S*_2_ and Li_2_S. Since 75% of the discharge capacity (1,254 mAh g^−1^) occurs in this conversion process, an enhanced reaction kinetics is beneficial for increasing the reversible capacity (Lim et al., 2019). However, since previous studies have shown that the absorption of LiPS by insulating interlayer is considered to be a “death zone” without transferring electrons during cycling (Hao et al., 2017). The fading trend in the battery with FeOOH interlayer mainly result from its low conductivity (10^−5^ S cm^−1^). Compared with relevant studies (Table S1), the introduction of this functional interlayer delivered better performance improvement for Li-S batteries.

The existence of functional interlayer can be used both as a conductive top current collector and as a physical barrier to polysulfide diffusion and lithium (Li) metal, debase the corrosion of Li metal. In case of rate performance (Figure S5b), CNTs@FeOOH-II composites exhibit high discharge capacity of 1292.6, 957.5, 802.3, and 630.8 mAh g^−1^ at various rates from 0.2 to 2C, which is more satisfactory than CNTs and FeOOH. In particular, when the rate was restored to 0.2C, the specific capacity of the battery returned to 972.5 mAh g^−1^. These results confirm that CNTs@FeOOH-II interlayer enhances the stability of S electrode. The presence of FeOOH in CNTs@FeOOH-II composite can enhance the rapid transformation of polysulfide ions and the hysteretic conversion kinetics of Li-S batteries, thus improving the rate capability of Li-S batteries.

**Figure 5:**
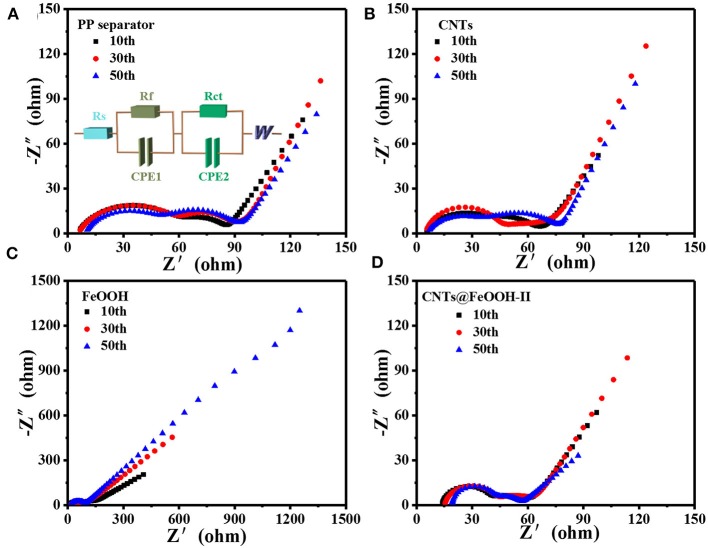
Nyquist plots of **(A)** PP separator, **(B)** CNTs, **(C)** FeOOH, and **(D)** CNTs@FeOOH-II.

The electrocatalytic effects of the different interlayers on PP separator were studied by electrochemical impedance spectroscopy (EIS). In Figure 5 shows the Nyquist plots when discharges up to 2.1 V after 10, 30, 50 cycles, respectively. Each plot consists of one oblique line in a low frequency region and one or two compressed semicircles in a medium and high frequency region. The corresponding equivalent circuit model is shown in Figure 5A. In the equivalent circuit, R_s_ represents the ohmic resistance of the reaction system; R_f_ is related to the resistance of the solid electrolyte interface (SEI), corresponding to a semicircle of the high frequency region; R_ct_ represents the charge transfer resistance, corresponding to a semicircle of the mid-frequency region, the diameter of the semicircle is the size of R_ct_, the larger the diameter, the greater the impedance, the more unfavorable to the high performance; CPE-double layer electrode/electrolyte capacitance; W characterizes the Warburg diffusion impedance of the electrode, which corresponds to an oblique line in the low frequency band, it characterizes the diffusion rate of Li^+^ in the material, the larger the slope, the better the high performance (Hu et al., 2018).

The fitting values of R_ct_ were exhibited in Table 2, the S electrode with CNTs@FeOOH-II interlayer endowed the lowest R_ct_ value after several charge-discharge cycles. The results are in good accordance with the results of the electrochemical cycle test. The conductivity of CNTs@FeOOH-II was measured by four-point probe method, which is about 4.6 S cm^−1^. High conductivity of CNTs@FeOOH-II may accelerate electron transfer and reduce electrochemical polarization. This further indicates that electrocatalytic materials with high conductivity and faster electron and ion transfer rates can improve the electrochemical performance of Li-S batteries.

**Table 2:** The comparison of Rct values of different interlayer and PP separator.

**Samples**	**10th**	**30th**	**50th**
PP separator	24.07	22.55	34.15
CNTs	22.46	18.2	37.12
FeOOH	48.49	28.68	54.96
CNTs@FeOOH-II	19.24	15.94	21.64

## Conclusion

In conclusion, FeOOH combined with CNTs with excellent catalytic ability is applied for high performance Li-S batteries. Compared to the pristine samples, the modified battery exhibited a good performance of 556 mAh g^−1^ at 3,200 mA g^−1^ for 350 cycles. CNTs@FeOOH-II plays the following main roles: (i) oxygen vacancies in FeOOH promote the rapid transformation of polysulfide ions, thus enhancing the reaction kinetics; (ii) the presence of FeOOH can effectively adsorb soluble polysulfides, which sluggishs the further diffusion to the anode; (iii) CNTs@FeOOH-II with high electric conductivity can be used as a “vice-electrode” to accelerate electron transfer and thus improve the rate capability. Therefore, for a high performance of Li-S battery, it is necessary to consider the high conductivity, adsorption, and fast conversion of LiPS.

## Data Availability Statement

All datasets generated for this study are included in the article/Supplementary Material.

## Author Contributions

YL, XL, and YH contributed conception and design of the study. YL organized the database, performed the statistical analysis, and wrote the first draft of the manuscript. All authors contributed to manuscript revision, read, and approved the submitted version.

## Conflict of Interest

LK, ZT, LS, and CZ were employed by the company Shaanxi Coal Chemical Industry Technology Research Institute Co., Ltd. The remaining authors declare that the research was conducted in the absence of any commercial or financial relationships that could be construed as a potential conflict of interest.
