# Supplementary material for: β-FeOOH Interlayer With Abundant Oxygen Vacancy Toward Boosting Catalytic Effect for Lithium Sulfur Batteries
Source: Front Chem. 2020 Apr 23;8:309. doi: 10.3389/fchem.2020.00309 (PMC7192061; doi:10.3389/fchem.2020.00309)
Supplement: Supplementary file 1 [file Data_Sheet_1.docx]

Supporting Information

**β-FeOOH interlayer with abundant oxygen vacancy towards boosting catalytic effect for lithium sulfur batteries**

Yingying Li^a^, Xifei Li^a,b,c*^, Youchen Hao^b^, Alibek Kakimov^b^, Dejun Li^a^, Qian Sun^d^, Liang Kou^e^, Zhanyuan Tian^e^, Le Shao^e^, Cheng Zhang^e^, Jiujun Zhang^b,f^, Xueliang Sun^a,b,d^

^a^Tianjin International Joint Research Centre of Surface Technology for Energy Storage Materials, Energy & Materials Engineering Centre, College of Physics and Materials Science, Tianjin Normal University, Tianjin 300387, China.

^b^Xi'an Key Laboratory of New Energy Materials and Devices, Institute of Advanced Electrochemical Energy & School of Materials Science and Engineering, Xi’an University of Technology, Xi’an 710048, Shaanxi, China.

^c^State Center for International Cooperation on Designer Low-carbon &Environmental Materials (CDLCEM), Zhengzhou University, 100 Kexue Avenue, Zhengzhou 450001, China.

^d^Department of Mechanical and Materials Engineering, University of Western Ontario, London, Ontario N6A 5B9, Canada.

^e^Shaanxi Coal Chem Ind Technol Res Inst Co Ltd, Xian, Shaanxi 710070, China.

^f^Department of Chemistry, College of Sciences/Institute for Sustainable Energy, Shanghai University, Shanghai, 200444, China

*Email: xfli2011@hotmail.com


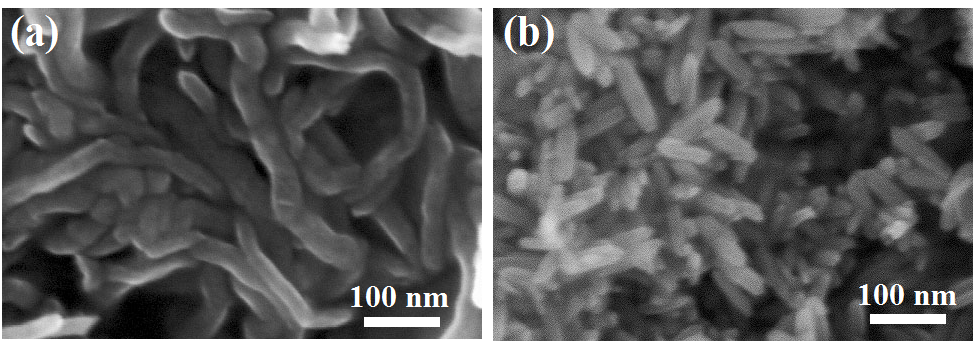


Figure S1. SEM images of (a) CNTs and (b) bare FeOOH sticks.


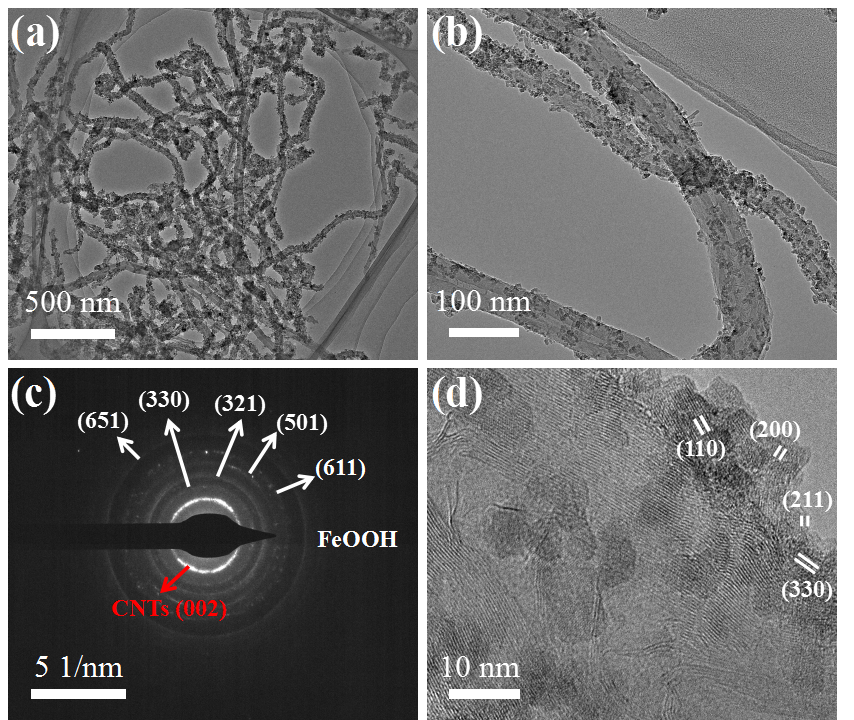


Figure S2. High and low-maganification TEM images of CNTs@FeOOH-II compounds and the corresponding SAED.


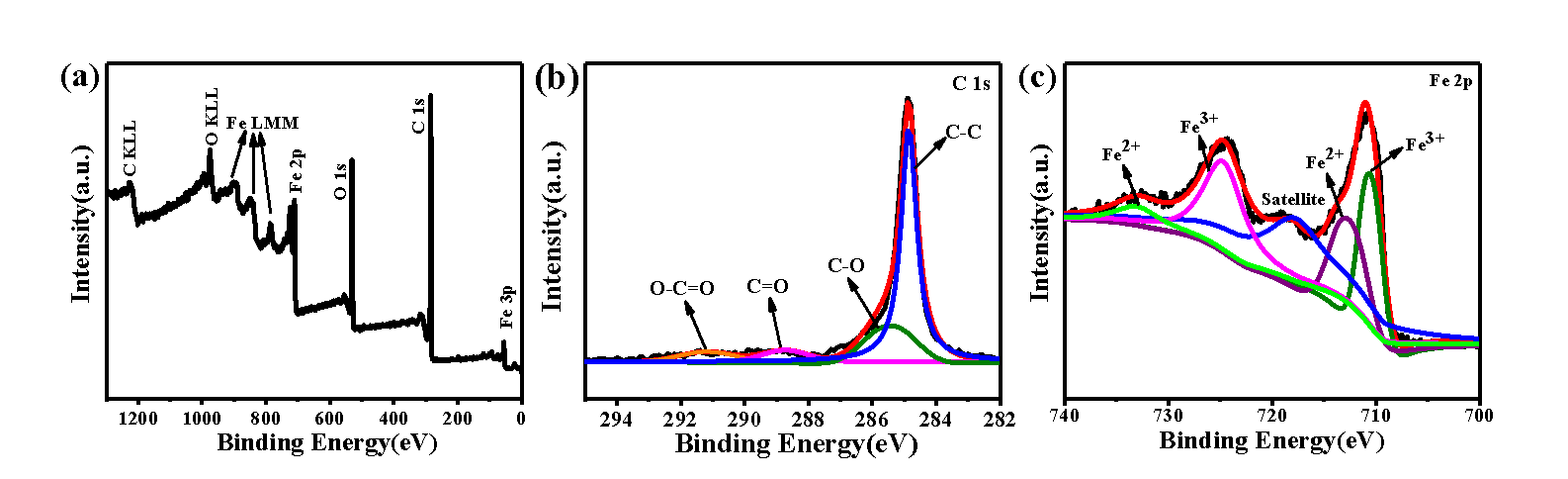


Figure S3. High-resolution XPS spectra of the (a) original XPS survey spectra, (b) C1s and (c) Fe 2p of CNTs@FeOOH-II composite.


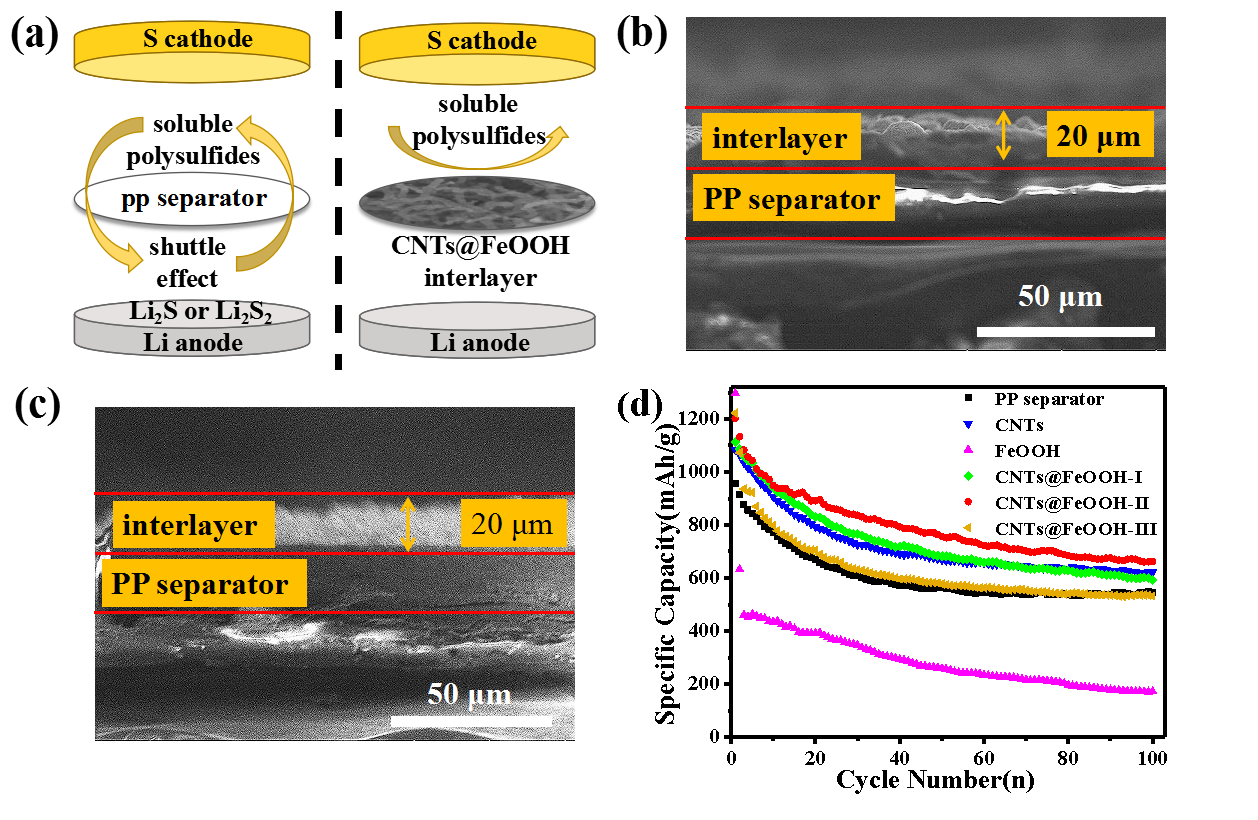


Figure S4. (a) Schematic diagram of conventional and improved Li-S cells; cross section of (b) CNTs@FeOOH-I and (c) CNTs@FeOOH-III interlayer; (d) Cycle performance of batteries with PP, CNTs, FeOOH, and CNTs@FeOOH separator, respectively, at 320 mA g^-1^.


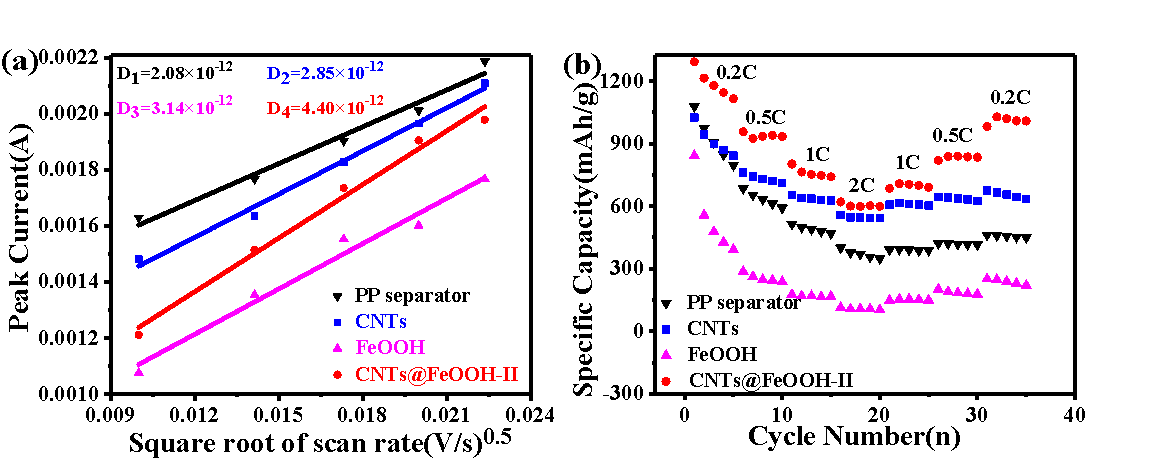


Figure S5. (a) The corresponding relationship between the square root of the scan rate V^0.5^ and peak current Ip of CNTs@FeOOH-II in a voltage range of 1.7–2.8 V vs. Li^+^/Li. (b) rate capability of four kinds of separator.

**Table S1** Comparison of the Li-S batteries with different functional interlayer, with results from this work and from the literature


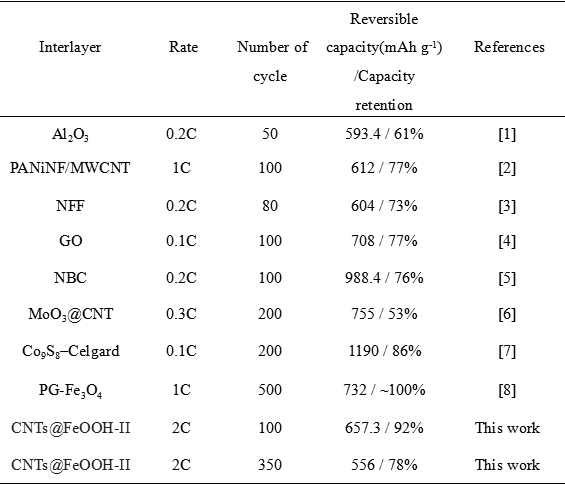


**References**

**[1]** Zhang Z; Lai Y; Zhang Z; Zhang K; Li J. Al_2_O_3_-coated porous separator for enhanced electrochemical performance of lithium sulfur batteries. Electrochimica Acta 2014, 129, 55-61.

**[2]** Chang C-H; Chung S-H; Manthiram A. Ultra-lightweight PANiNF/MWCNT-functionalized separators with synergistic suppression of polysulfide migration for Li–S batteries with pure sulfur cathodes. Journal of Materials Chemistry A 2015, 3, 18829-18834.

**[3]** Jia-Qi; Huang T-ZZ; Qiang Zhang; Hong-Jie Peng; Cheng-Meng Chen; and Fei Wei. Permselective Graphene Oxide Membrane for Highly Stable and Anti-Self-Discharge Lithium Sulfur Batteries. ACS Nano 2015, 9 3002–3011.

**[4]** Zhang K; Qin F; Fang J; Li Q; Jia M; Lai Y; Zhang Z; Li J. Nickel foam as interlayer to improve the performance of lithium–sulfur battery. Journal of Solid State Electrochemistry 2013, 18, 1025-1029.

**[5]** Cai W; Li G; Zhang K; Xiao G; Wang C; Ye K; Chen Z; Zhu Y; Qian Y. Conductive Nanocrystalline Niobium Carbide as High-Efficiency Polysulfides Tamer for Lithium-Sulfur Batteries. Advanced Functional Materials 2018, 28.

**[6]** Luo L; Qin X; Wu J; Liang G; Li Q; Liu M; Kang F; Chen G; Li B. An interwoven MoO_3_@CNT scaffold interlayer for high-performance lithium–sulfur batteries. Journal of Materials Chemistry A 2018, 6, 8612-8619.

**[7]** He J; Chen Y; Manthiram A. Vertical Co_9_S_8_ Hollow Nanowall Arrays Grown on A Celgard Separator as A Multifunctional Polysulfide Barrier for High-Performance Li–S Batteries. Energy & Environmental Science 2018, 11, 2560-2568.

**[8]** He J; Luo L; Chen Y; Manthiram A. Yolk-Shelled C@Fe_3_O_4_ Nanoboxes as Efficient Sulfur Hosts for High-Performance Lithium-Sulfur Batteries. Adv Mater 2017, 29.
